# Antidiabetic and Hepatorenal Protective Effects of *Annona reticulata* Linn. Leaf Extract Alone and in Combination With Glibenclamide in Alloxan‐Induced Diabetic Rats

**DOI:** 10.1155/tswj/2052797

**Published:** 2026-07-28

**Authors:** Tasnia Binte Bari Kabbo, Fahim Shahrier Rahman, Md. Sohel Rana, Pritesh Ranjan Dash

**Affiliations:** ^1^ Department of Pharmacy, Jahangirnagar University, Savar, Dhaka, Bangladesh, juniv.edu; ^2^ Department of Pharmacy, ASA University Bangladesh, Dhaka, Bangladesh, asaub.edu.bd

**Keywords:** *Annona reticulata* Linn, antidiabetic activity, extract, lipid profile, liver-renal protective activity

## Abstract

The pharmacological properties of *Annona reticulata* Linn. (*Annonaceae*) leaves, as reported by some tribal tribes, were investigated in this study. The antidiabetic and liver‐renal protective potentials of methanolic leaf fraction and its combination with glibenclamide were evaluated in rats having alloxan‐induced diabetes at doses of 200 and 400 mg/kg body weight; demonstrated notable antidiabetic potential which was dose‐dependent. Following 21 days course of treatment, methanol leaf fraction (400 mg/kg) and combination of extract (200 mg/kg) along with glibenclamide (2.5 mg/kg) exhibited fasting blood glucose levels of 5.25 ± 0.06 mmol/L and 6.02 ± 0.07 mmol/L; whereas standard drug glibenclamide (5 mg/kg) showed fasting blood glucose level of 5.91 ± 0.09 mmol/L. Furthermore, the diabetic rats in extract group (400 mg/kg dosage) and in combination group showed body weight reduction values of 14.67 and 16.5 gm; which was lower than the value obtained group of rats treated with glibenclamide alone (21.83 gm). Additionally, the extract and its combination with glibenclamide were found to have significant protective impacts on the kidney and liver (*p* < 0.001) and to aid in the maintenance of lipid profile even in the presence of diabetes; these effects were comparable with the outcomes obtained from standard drug. Although diabetic rats demonstrated elevated liver and kidney markers levels, the groups of mice treated with methanolic leaf extract at both 200 and 400 mg/kg dosages exhibited notable maintenance of both liver and kidney markers levels. Furthermore, combination of methanol extract and glibenclamide showed serum ALT, AST, bilirubin and creatinine, BUN values of 15.20 ± 1.58 IU/L, 10.73 ± 0.45 IU/L, 3.60 ± 0.22 * μ*mol/L; and 0.73 ± 0.03 mg/dL, 18.13 ± 2.13 mg/dL (*p* < 0.001); which were markedly lower than the values obtained from diabetic control rats. Moreover, both methanolic leaf extract and standard drug glibenclamide as well as their combination exhibited beneficial effect in the maintenance of serum TC, TG, LDL, and HDL levels. Methanolic extract at 400 mg/kg dosage and the combination demonstrated serum TC, TG, LDL, and HDL values of 84.33 ± 3.00 mg/dL, 62.32 ± 3.13 mg/dL, 42.67 ± 3.01 mg/dL, 37.43 ± 6.47 mg/dL; and 80.43 ± 3.53 mg/dL, 65.20 ± 6.29 mg/dL, 44.50 ± 2.88 mg/dL, 43.52 ± 3.33 mg/dL (*p* < 0.001), respectively. Additionally, the extract′s potential components that may have played crucial roles in achieving these bioactivities were identified using the reports from the GC‐MS analysis.

## 1. Introduction

GC‐MS technique identifies the components in a sample [1]. GC‐MS analysis is frequently performed on plant extracts to identify and quantify volatile and semivolatile organic components. This approach yields a thorough chemical profile of the plant by contrasting its chemical profile with an existing library of chemicals. It facilitates the process of identifying plant parts and comprehending the biological roles of the substances derived from plants [2, 3]. Diabetes mellitus is not a single disease; rather, it is a collection of metabolic illnesses. Insufficient production of insulin, inadequate target cell response to insulin, and inadequate secretion of the insulin hormone can lead to diabetes [4]. Adverse effects are a common side effect of contemporary oral hypoglycemic medications. Thus, alternative therapy is needed, and a move toward various indigenous plant and herbal compositions is urgently needed. The use of traditional medicines in diabetes treatment has shown promise [5]. Herbal medicine formulations that are part of the traditional medical systems have long been recognized as liver‐protective compounds. Together with other benefits, the hepatoprotective potential of all the following plants is enormous. Due to their ability to prevent and treat liver problems, a variety of edible herbs have also been approved. Through a variety of techniques, they have demonstrated their hepatoprotective activity [6]. It is now known that nephrotoxicity from drugs used as treatments for other conditions, such as pre‐renal and postrenal diseases, increases the likelihood of developing acute and chronic kidney disorders. To avoid the side effects of medication, alternative methods of treating certain conditions have been investigated. Many ailments have traditionally been treated with herbal treatments [7]. Traditionally significant plant, *Annona reticulata* Linn., was selected for assessment of its antidiabetic and liver‐renal protective properties since it has been used ethnomedically to treat a range of ailments. Although some researches have investigated the antidiabetic and liver‐renal protective properties of *A. reticulata* Linn. extracts, this was the first time the probable phytoconstituents leading to these bioactivities were identified. Furthermore, the antidiabetic and liver‐renal protective potentials of the combination of glibenclamide and methanol leaf extract were evaluated for the first time via this study.

## 2. Materials and Methods

### 2.1. Preparation of Plant Extract

Plant material (dried) weighing 630 g was sealed in dark‐colored flasks with 6.5 L of methanol. Twenty‐four hours later, the filtration of infusions was conducted. The process was further repeated 48 h later. Under vacuum, drying of supernatants in rotary evaporator (40°C) was conducted. Extraction was thought to be complete when the plant materials were verified to be exhausted. The plant material was extracted, dried, and submerged in 1 L of methanol in a sealed container for 7 days, with regular shaking and stirring. A fresh cotton bed filter was used to filter the extract. A sticky crude extract concentration was created by drying the resultant filtrates at 40^°^C ± 2^°^C. The extracted materials were labeled appropriately and stored in sterile sample containers at 4°C [8, 9].

### 2.2. GC‐MS Analysis of the Extract From *A. reticulata* Linn. Leaves

For performing GC‐MS analysis, Shimadzu GCMS‐TQ8040 was utilized. The analytes were successfully separated and identified by utilizing Helium (carrier gas). According to the GC parameters, the column oven was set to 50°C, ramped up to 200°C, and lastly reached 300°C. The hold times were 1, 2, and 7 min, respectively. The injection was conducted (250°C) with a sampling duration of 1 min, flow control mode set to pressure at 53.5 kPa, and total flow of 11 mL/min. Under MS circumstances, 230°C and 250°C were the temperatures of the ion source and contact. This configuration provides excellent sensitivity and specificity for identifying and quantifying volatile and semivolatile substances in complex plant extracts by comparing them to library compounds [9].

### 2.3. In Vivo Studies

#### 2.3.1. Experimental Animals

Wister albino rats (120–230 g) and Swiss albino mice (25–30 g) were taken for this investigation. Test animals were gathered from the department′s own animal house. *A. reticulata* Linn. leaf extract was studied pharmacologically in the Jahangirnagar University Pharmacology Laboratory. In polypropylene cages with proper dark‐light cycle, relative humidity (RH 55*%* ± 5*%*), and temperature of 25^°^C ± 2^°^C, test animals were kept. The rats and mice were given ICDDR, B. pelletized mouse feed and had unrestricted access to water. The instructions issued by Jahangirnagar University′s animal ethics committee were followed in every action involving the care of animals [Ref No: BBEC, JU/M 2024/11 (137); Date of Approval: 19‐11‐2024].

#### 2.3.2. Assay of In Vivo Acute Toxicity Study

To ascertain the safety profile of the plant extract intended for human consumption, an acute oral toxicity study was conducted following the OECD Guideline 423 (OECD, 2001), Acute Toxic Class Method, as described by Harizal et al. (2010). Healthy albino mice were divided into three groups, each consisting of five mice. All animals were kept starving through the night (with free access to water) before administration of plant extract. Then, the experimental animals were given methanol leaf extract of *A. reticulata* Linn. at progressively higher doses, such as 1, 2, and 4 gm/kg via intragastric tube for selecting the optimal dosage. Following administration, the animals were closely observed individually for signs of toxicity, behavioral changes, and mortality, with special attention given during the first 4 h and periodically thereafter. The animals were further monitored daily for a total period of 14 days to detect any delayed toxic effects [10, 11].

#### 2.3.3. Evaluation of In Vivo Antidiabetic Potential and Protective Effect on the Liver and Kidney

Using rats having alloxan‐induced diabetes, this study assessed antidiabetic activity of *A. reticulata* Linn. methanolic leaf fraction and its combination with glibenclamide. By comparing antidiabetic responses of animals given test samples (*A. reticulata* Linn. leaf extract and extract′s combination with glibenclamide) to animals given distilled water (negative control) and standard medication (positive control), the effectiveness of the antidiabetic potential was evaluated. In order to illustrate the treatment outcomes of the combination and for comparison of outcomes obtained from combination with individual results of test fraction and medicine, the extract and standard medication combination was evaluated. Additionally, this was done to test if the combination could lessen the side effects of conventional therapy. Since diabetes gradually damages the liver and kidney, tests of several serum biochemical parameters were also done to evaluate the extract′s and combination′s protective effect on these organs. To demonstrate the impact on the kidney, liver, and pancreas, histopathological assessment was performed.

#### 2.3.4. Induction of Diabetes

Alloxan (150 mg/kg) was administered via injecting intraperitoneally to 40 rats. After they had been fasting for 18 h. The alloxan was dissolved in 5% dextrose saline solution. Hypoglycemic shock was prevented by providing unlimited food and water following the injection. The onset of diabetes was verified 48 h after injection. Rats having FBG levels above 11.1 mmol/L were chosen [12–16].

#### 2.3.5. Treatment Protocol

Thirty rats were divided into six groups, each group consisting of five rats. Each rat received a tail number after being weighed. Five normal control rats made up Group I. Five rats with diabetes produced by alloxan make up the remaining groups. Animals in Group I, which were normal controls, were given 10 mL/kg of distilled water; animals in Group II, which were diabetics caused by alloxan, were given the same. Glibenclamide (5 mg/kg) was given via the oral route to Group III diabetic rats. Animals with diabetes caused by alloxan in Group IV and V received oral dosages of 200 and 400 mg/kg of methanol leaf extract. Combination of glibenclamide (2.5 mg/kg) and methanolic fraction (200 mg/kg) was administered orally to Group VI rats, who were diabetics due to alloxan. Every animal in all groups received therapy according to the given schedule for a total of 21 days. In order to measure the blood glucose level using an electronic glucometer, blood samples were taken 1 h after the medicine was administered on Days 1, 7, 14, and 21. End tail vein cutting was used to get blood samples, and a one‐touch electronic glucometer was used to measure blood glucose levels. Additionally, on Days 1, 7, 14, and 21, the rats′ body weight was carefully recorded [12–16]. Percentage (%) of body weight changes was calculated using standard formula [16].

#### 2.3.6. Evaluation of Antidiabetic Activity and Protective Effect on Liver and Kidney

The evaluation of antidiabetic activity and protective effect on liver and kidney is described as follows:

▪ Assessment of biochemical parameters: 21 days later, rats underwent overnight fasting and were then sacrificed. Separation of serum from blood (obtained from the retro‐orbital plexus) using a microcapillary technique was conducted. After that, utilizing a commercial kit, estimation of serum triglycerides (TG), total cholesterol (TC), HDL, LDL, creatinine, blood urea nitrogen, ALT, AST, bilirubin, and random blood glucose level was carried out [13, 16].
▪ Histopathological assessment: After sacrifice, removal of pancreas, liver, and kidney was done and kept in 10% formalin solution. Then, cutting of segments (5 *μ* thick) was performed. Then, staining was conducted by utilizing hematoxylin and eosin [15, 16].

Statistical analysis was performed using SPSS for Windows (Version 27.0). Data were expressed as mean ± SEM. Differences among groups were analyzed using ANOVA, followed by post hoc multiple comparison test to determine intergroup differences. Exact *p* values were calculated and reported, and a value of *p* < 0.05 was considered statistically significant.

## 3. Result and Discussion

### 3.1. GC‐MS Analysis Report of Methanol Extract of *A. reticulata* Linn. Leaves

According to the library search report, there were 43 components in methanolic leaf fraction from *A. reticulata* Linn. (Table 1).

**Table 1:** Components detected by GC‐MS analysis in *Annona reticulata* Linn.′s crude aqueous extract.

Serial no.	Compound name	Molecular formula and molecular weight	Area
1	Hexadecanoic acid, methyl ester	C_17_H_34_O_2_; 270.45 g/mol	506819
2	Tridecanoic acid, 12‐methyl‐, methyl ester	C_15_H_30_O_2_; 242.40 g/mol	506819
3	Tridecanoic acid, methyl ester	C_14_H_28_O_2_; 228.37 g/mol	506819
4	Decanoic acid, methyl ester	C_11_H_22_O_2_; 186.29 g/mol	506819
5	Methyl tetradecanoate	C_15_H_30_O_2_; 242.39 g/mol	506819
6	9,12‐Octadecadienal	C_18_H_32_O; 264.44 g/mol	43473
7	Dichloroacetic acid, dodec‐9‐ynyl	C_14_H_18_Cl_2_O_2_; 289.18 g/mol	43473
8	E,E‐1,9,17‐docasatriene	C_22_H_40_; 304.54 g/mol	43473
9	9‐Dodecyn‐1‐ol	C_12_H_22_O; 182.30 g/mol	43473
10	3‐Tetradecyne	C_14_H_28_; 196.36 g/mol	43473
11	10‐Undecyn‐1‐ol	C_11_H_22_O; 170.29 g/mol	43473
12	13‐Tetradece‐11‐yn‐1‐ol	C_14_H_28_O; 212.36 g/mol	43473
13	E‐1,6‐Undecadiene	C_11_H_20_; 152.27 g/mol	43473
14	11,14,17‐Eicosatrienoic acid, methyl ester	C_21_H_34_O_2_; 318.48 g/mol	557717
15	Cis,cis,cis‐7,10,13‐hexadecatrienal	C_16_H_26_O; 234.37 g/mol	557717
16	5‐Pentadecen‐7‐yne, (Z)‐	C_15_H_28_; 208.37 g/mol	557717
17	3‐Heptadecen‐5‐yne, (Z)‐	C_17_H_32_; 236.43 g/mol	557717
18	1‐Tetradecen‐3‐yne	C_14_H_26_; 194.35 g/mol	557717
19	Phytol	C_20_H_40_O; 296.52 g/mol	257362
20	3,4‐Dimethylcyclohexanol	C_8_H_16_O; 128.21 g/mol	257362
21	1,7‐Octadien‐3‐ol, 2,6‐dimethyl‐	C_10_H_18_O; 154.24 g/mol	257362
22	Oxirane, decyl‐	C_12_H_24_O; 184.31 g/mol	257362
23	Methyl stearate	C_19_H_38_O_2_; 298.49 g/mol	278138
24	Eicosanoic acid, methyl ester	C_21_H_42_O_2_; 326.55 g/mol	278138
25	Octadecanoic acid, 17‐methyl‐, methyl ester	C_20_H_40_O_2_; 312.52 g/mol	278138
26	Methyl tetradecanoate	C_15_H_30_O_2_; 242.39 g/mol	278138
27	13‐Docosenamide, (Z)‐	C_22_H_41_NO; 335.56 g/mol	133359
28	9‐Octadecenamide, (Z)‐	C_18_H_35_NO; 281.47 g/mol	133359
29	8‐Methyl‐6‐nonenamide	C_10_H_21_NO; 171.28 g/mol	133359
30	3‐Isopropoxy‐1,1,1,5,5,5‐hexamethyl‐3‐(trimethyl)pentane	C_13_H_30_O; 202.39 g/mol	13558
31	Thymol, TMS derivative	C_13_H_21_OSi; 221.39 g/mol	13558
32	1,2‐Bis(trimethylsilyl)benzene	C_12_H_22_Si_2_; 222.42 g/mol	13558
33	1‐Isopropoxy‐5‐propyl‐2,3‐bis(trimethylsilyl)benzene	C_18_H_34_OSi_2_; 322.62 g/mol	13558
34	2‐(N,N’,N’‐Trimethylhydrazino)‐1,3‐benzothiazole	C_10_H_14_N_3_S; 208.31 g/mol	13558
35	1‐hexadecanesulfonamide, N‐(3‐aminopropyl)‐	C_19_H_41_N_2_O_2_S; 361.61 g/mol	13558
36	3,5‐bis(trimethylsilyl)cyclohepta‐2,4,6‐trien‐1‐one	C_13_H_24_OSi_2_; 252.50 g/mol	13558
37	Trisiloxane, 1,1,1,5,5,5‐hexamethyl‐3,3‐bis(trimethylsilyl)oxy‐	C_12_H_36_O_5_Si_5_; 400.86 g/mol	13558
38	Trimethylsilyl‐di(timethylsiloxy)‐silane	C_9_H_27_O_2_Si_4_; 279.67 g/mol	13558
39	Pterin‐6‐carboxylic acid	C_7_H_5_N_5_O_3_; 207.16 g/mol	26957
40	1,4‐Bis(trimethylsilyl)benzene	C_12_H_22_Si_2_; 222.48 g/mol	26957
41	Cholesterol 3‐O‐[[2‐acetoxy]ethyl]‐	C_31_H_52_O_2_; 455.72 g/mol	26957
42	Androstane‐11,17‐dione, 3‐[(trimethylsilyl)oxy	C_22_H_34_O_2_Si; 358.58 g/mol	26957
43	1,5,6,7‐Tetramethylbicyclo[3.2.0]hept‐6‐en‐3‐yl	C_11_H_19_; 151.26 g/mol	26957

**Figure 1:**
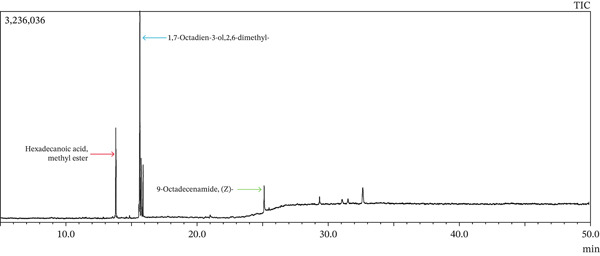
Total ionic chromatogram of *Annona reticulata* Linn. crude methanol extract.

Among these compounds 1,7‐octadien‐3‐ol, 2,6‐dimethyl‐ is reported to possess significant antidiabetic and renal‐protective effect [17–19]. Additionally, hexadecanoic acid‐methyl ester is reported to have strong hepatoprotective activity [20, 21]. Moreover, 9‐octadecenamide (z)‐ might help control lipids and cholesterols since it has hypolipidemic potential, as per reports [22, 23] (Figure 1).

### 3.2. In Vivo Studies

#### 3.2.1. In Vivo Evaluation of Acute Toxicity

Experimental animals were given methanol extract of *A. reticulata* Linn. leaves at doses of 1, 2, and 4 gm/kg. Following 14 days observation period, no signs of death, morbidity, or abnormal behavioral changes were observed in *A. reticulata* Linn.′s methanol leaf extract, indicating that the extract was well tolerated. Shivanna et al. validated in a study on the assessment of *A. reticulata* Linn. extracts′ acute toxicity that there is no indication of either acute or delayed toxicity in experimental animals. Therefore, the extract can be safely given to experimental animals [24].

#### 3.2.2. Evaluation of In Vivo Antidiabetic Potential and Protective Effect on the Liver and Kidney

**Figure 2:**
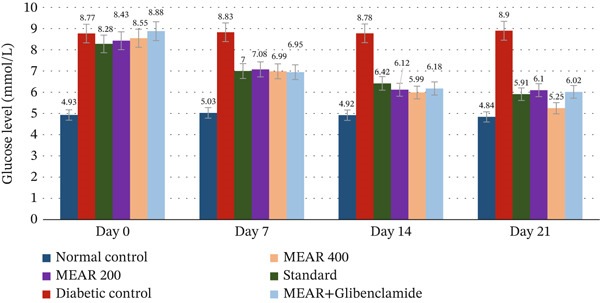
Effect of standard, methanol extract and their combination on FBG level after 21 days treatment.

Rats having alloxan‐induced diabetes underwent treatment for 21 days to evaluate the test samples′ antidiabetic activities. Regular monitoring of blood glucose level was conducted since it can be used as an indicator of the test samples′ antidiabetic efficacy. Rats′ normal FBG level range from 4.4 to 4.9 mmol/L [25]. The normal control group′s FBG levels on Day 0, 7, 14, and 21 were 4.93 ± 0.16 mmol/L, 5.03 ± 0.16 mmol/L, 4.92 ± 0.06 mmol/L, and 4.84 ± 0.15 mmol/L, respectively. These rats have fasting blood glucose levels that fall within the normal range because they are healthy. FBG levels of diabetic control group were much higher when compared with normal, measuring 8.77 ± 0.21 mmol/L, 8.83 ± 0.17 mmol/L, 8.78 ± 0.13 mmol/L, and 8.90 ± 0.05 mmol/L on days 0, 7, 14, and 21, respectively. Glibenclamide‐treated standard group′s blood glucose levels decreased over the course of treatment; on Days 0, 7, 14, and 21, the values were 8.28 ± 0.16 mmol/L, 7.00 ± 0.07 mmol/L (*p* < 0.001), 6.42 ± 0.15 mmol/L (*p* < 0.001), and 5.91 ± 0.09 mmol/L (*p* < 0.001). These numbers, however, remain above the normal range. The methanol extract demonstrated good diabetic control as well. Accordingly, on Days 0, 7, 14, and 21, the group administered 400 mg/kg of methanolic fraction demonstrated fasting blood glucose level values of 8.55 ± 0.26 mmol/L, 6.99 ± 0.10 mmol/L (*p* < 0.001), 5.99 ± 0.05 mmol/L (*p* < 0.001), and 5.25 ± 0.06 mmol/L (*p* < 0.001). Additionally, the 200 mg/kg methanol extract treated group also had good results, with FBG levels on Days 0, 7, 14, and 21 measuring 8.43 ± 0.21 mmol/L, 7.08 ± 0.05 mmol/L (*p* < 0.001), 6.12 ± 0.07 mmol/L (*p* < 0.001), and 6.10 ± 0.07 mmol/L (*p* < 0.001). Furthermore, animals treated with a combination of glibenclamide and methanol extract showed good antidiabetic potential (fasting blood glucose level values of 8.88 ± 0.07 mmol/L, 6.95 ± 0.04 mmol/L (*p* < 0.001), 6.18 ± 0.10 mmol/L (*p* < 0.001), and 6.02 ± 0.07 mmol/L (*p* < 0.001) on Days 0, 7, 14, and 21, respectively). Regardless of the group, it is evident that all experimental animals had extremely high blood glucose levels on Day 0 due to the fact that treatments had begun on this day and measurements had been completed prior to treatment starting. The blood glucose level then steadily dropped (Table 2). For the extract, a definite dose‐dependent rise in antidiabetic efficacy is seen (Figure 2). In addition, it is apparent that methanol leaf extract alone and its combination with glibenclamide demonstrated similar antidiabetic outcomes in our investigation. In the order shown below, the test samples′ antidiabetic activity declined:

**Table 2:** Effect of standard, methanol extract and their combination on FBG level (mmol/L) after 21 days treatment.

Group	Day 0	Day 7	Day 14	Day 21
Normal control	4.93 ± 0.16	5.03 ± 0.16	4.92 ± 0.06	4.84 ± 0.15
Diabetic control	8.77 ± 0.21	8.83 ± 0.17	8.78 ± 0.13	8.90 ± 0.05
Standard	8.28 ± 0.16	7.00 ± 0.07^∗∗∗^	6.42 ± 0.15^∗∗∗^	5.91 ± 0.09^∗∗∗^
MEAR 200	8.43 ± 0.21	7.08 ± 0.05^∗∗∗^	6.12 ± 0.07^∗∗∗^	6.10 ± 0.07^∗∗∗^
MEAR 400	8.55 ± 0.26	6.99 ± 0.10^∗∗∗^	5.99 ± 0.05^∗∗∗^	5.25 ± 0.06^∗∗∗^
MEAR + glibenclamide	8.88 ± 0.07	6.95 ± 0.04^∗∗∗^	6.18 ± 0.10^∗∗∗^	6.02 ± 0.07^∗∗∗^

*Note:* Standard is glibenclamide.

Abbreviation: MEAR, methanol extract of *Annona reticulata* Linn.

^∗∗∗^ *p* < 0.001 (very high significance) against control ; Standard is Glibenclamide.

MEAR 400 > standard > MEAR + glibenclamide > MEAR 200.

**Figure 3:**
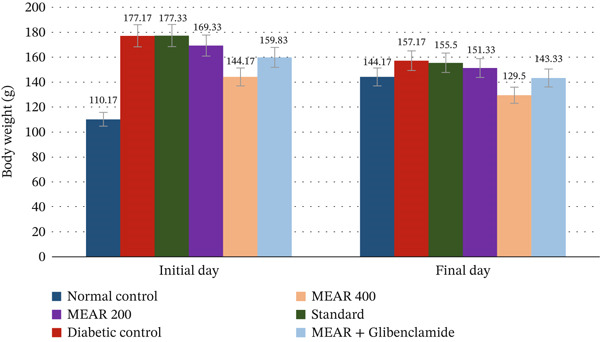
Effect of standard, methanol extract and their combination on body weights after 21 days treatment.

During the treatment of diabetes, the change in body weight was also monitored carefully. At the start of treatment, body weights of experimental rats were measured. Also, measurement of body weights was done at regular intervals during the entire course of treatment to determine if there was any abnormal change in body weight. For the normal control group, the initial body weight was 110.17 ± 1.58 g at Day 0 and the final body weight was 144.17 ± 2.08 g at Day 21; demonstrating 30.86% gain in body weight. As these rats were normal, their body weights were increased. For diabetic control rats, initial body weight was 177.17 ± 4.35 g at Day 0 and final body weight was 157.17 ± 5.94 g at Day 21, illustrating 11.29% loss in body weight; the rats of this group exhibited a reduction in body weight. The standard group that was given glibenclamide also showed a decrease in body weight starting with 177.33 ± 17.81 g and ending with 155.50 ± 13.61 g; showing 12.31% loss in body weight. The groups that were treated with methanol extract and its combination with glibenclamide also demonstrated gradual decrease in body weight with time. Groups that were administered methanolic leaf fraction at 200 and 400 mg/kg doses exhibited initial body weight values of 169.33 ± 8.45 g, 144.17 ± 3.95 g and final body weight values of 151.33 ± 7.42 g, and 129.50 ± 3.10 g, respectively. In addition, the 200 and 400 mg/kg dosages of methanol extract exhibited 10.63% and 10.17% reduction in body weight. The combination of methanol extract and glibenclamide treated group showed initial body weight of 159.83 ± 15.78 g at day 0 and final body weight of 143.33 ± 12.37 g after 21 days of treatment; showing 10.32% reduction in body weight (Table 3). Therefore, the body weight reduction was lower in the groups treated with methanol extract alone and in combination with glibenclamide (Figure 3).

**Table 3:** Effect of standard, methanol extract and their combination on body weights after 21 days treatment.

Group	Initial BW (g) (*m* *e* *a* *n* ± *S* *E* *M*)	Final BW (g) (*m* *e* *a* *n* ± *S* *E* *M*)	Percentage (%) of body weight changes
Normal control	110.17 ± 1.58	144.17 ± 2.08	+30.86%
Diabetic control	177.17 ± 4.35	157.17 ± 5.94	−11.29%
Standard	177.33 ± 17.81	155.50 ± 13.61	−12.31%
MEAR 200	169.33 ± 8.45	151.33 ± 7.42	−10.63%
MEAR 400	144.17 ± 3.95	129.50 ± 3.10	−10.17%
MEAR + glibenclamide	159.83 ± 15.78	143.33 ± 12.37	−10.32%

*Note:* Standard is glibenclamide. The (+) sign indicates increase in body weight and (–) sign indicates decrease in body weight.

Abbreviations: BW, body weight; MEAR, methanol extract of *Annona reticulata* Linn.

**Figure 4:**
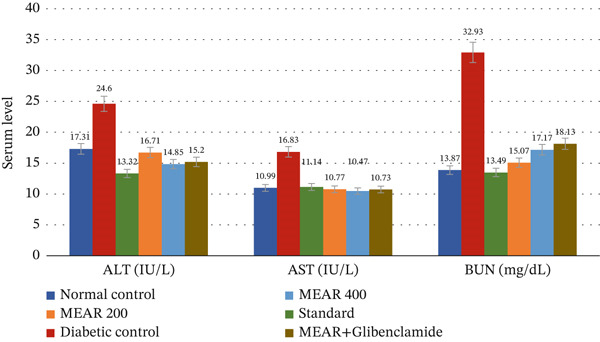
Effect of standard, methanol extract and their combination on ALT, AST, and BUN level after 21 days treatment.

Diabetes can have a negative impact on the liver because it is a metabolic disease and the liver is the primary organ of metabolism. To evaluate the test samples′ protective effect on the liver, a number of liver markers were examined following the course of treatment. Following dissection, serum was extracted from the test animals′ blood, and the levels of many significant liver markers were determined. According to Hasan et al., rats′ normal ranges for serum ALT, AST, and bilirubin are 6–45 IU/L, 6–30 IU/L, and 3–3.2 *μ*mol/L, respectively [26]. Serum ALT, AST, and bilirubin levels in the normal control group were 17.31 ± 2.55 IU/L, 10.99 ± 1.05 IU/L, and 1.97 ± 0.33 * μ*mol/L, in due order. Serum ALT, AST, and bilirubin measurements in the diabetic control group were much higher if compared with the normal range, with values of 24.60 ± 1.80 IU/L, 16.83 ± 1.46 IU/L, and 5.44 ± 0.61 * μ*mol/L. In contrast, the standard group that was given glibenclamide showed positive effects on the liver, with three liver markers measuring 13.32 ± 2.43 IU/L (*p* < 0.001), 11.14 ± 1.15 IU/L (*p* < 0.001), and 3.10 ± 0.10 * μ*mol/L (*p* < 0.001). In addition, the level of liver markers can also be effectively maintained by the methanol extract. Animals that were administered 400 mg/kg of methanolic leaf fraction had ALT, AST, and bilirubin levels of 14.85 ± 0.69 IU/L (*p* < 0.001), 10.47 ± 0.99 IU/L (*p* < 0.001), and 3.09 ± 0.10 * μ*mol/L (*p* < 0.001). Moreover, rats which received 200 mg/kg of methanolic fraction had three liver marker values of 16.71 ± 1.78 IU/L (*p* < 0.001), 10.77 ± 0.69 IU/L (*p* < 0.001), and 3.23 ± 0.26 * μ*mol/L (*p* < 0.001), in due order. Lastly, when methanol extract was combined with glibenclamide, the liver marker values were 15.20 ± 1.58 IU/L (*p* < 0.001), 10.73 ± 0.45 IU/L (*p* < 0.001), and 3.60 ± 0.22 * μ*mol/L (*p* < 0.001), in due order (Table 4). Thus, it may be concluded that the extract has the ability to shield the liver from the harm that diabetes causes (Figure 4).

**Table 4:** Effect of standard, methanol extract and their combination on ALT, AST, and bilirubin level after 21 days treatment.

Group	ALT (IU/L)	AST(IU/L)	Bilirubin (*μ*mol/L)
Normal control	17.31 ± 2.55	10.99 ± 1.05	1.97 ± 0.33
Diabetic control	24.60 ± 1.80	16.83 ± 1.46	5.44 ± 0.61
Standard	13.32 ± 2.43^∗∗∗^	11.14 ± 1.15^∗∗∗^	3.10 ± 0.10^∗∗∗^
MEAR 200	16.71 ± 1.78^∗∗∗^	10.77 ± 0.69^∗∗∗^	3.23 ± 0.26^∗∗∗^
MEAR 400	14.85 ± 0.69^∗∗∗^	10.47 ± 0.99^∗∗∗^	3.09 ± 0.10^∗∗∗^
MEAR + glibenclamide	15.20 ± 1.58^∗∗∗^	10.73 ± 0.45^∗∗∗^	3.60 ± 0.22^∗∗∗^

*Note:* Standard is glibenclamide.

Abbreviation: MEAR, methanol extract of *Annona reticulata* Linn.

^∗∗∗^ *p* < 0.001 (very high significance) against control; Standard is Glibenclamide.

**Figure 5:**
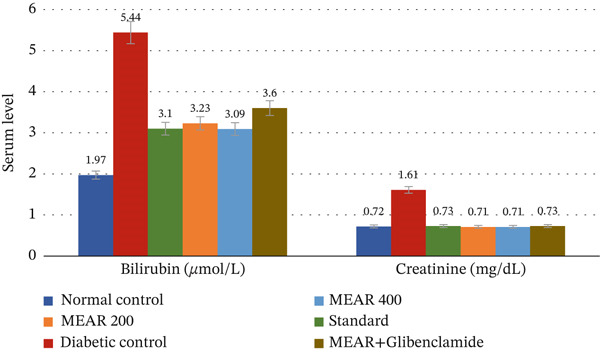
Effect of standard, methanol extract and their combination on bilirubin and creatinine level after 21 days treatment.

Kidney′s main function is excretion of wastes and extra fluid from the body. As diabetes is a metabolic disorder, it can cause harm to the kidney in many ways, most commonly by damaging the blood vessels of the kidney. Therefore, after the treatment period two important kidney markers were tested to evaluate whether the test samples can have a beneficial effect on the kidney despite the presence of diabetes or not. The normal ranges of BUN and creatinine (in serum) for rats are 15–30 mg/dL and 0.4–0.8 mg/dL, respectively [27, 28]. For the normal control group, obtained serum creatinine and BUN levels were 0.72 ± 0.09 mg/dL and 13.87 ± 2.57 mg/dL. Whereas, for the diabetic control group the values were 1.61 ± 0.35 mg/dL and 32.93 ± 3.74 mg/dL; these values were much higher than the normal range. The glibenclamide treated standard group showed serum creatinine and BUN levels of 0.73 ± 0.05 mg/dL (*p* < 0.001) and 13.49 ± 3.64 mg/dL (*p* < 0.001); these values were maintained in the normal range for rats. Methanolic leaf fraction exhibited good control over the kidney markers both alone and in combination with glibenclamide, which indicates that the extract can have a beneficial effect on the kidney even in the presence of diabetes. Methanolic leaf fraction at 200 and 400 mg/kg dosages exhibited serum creatinine and BUN values of 0.71 ± 0.09 mg/dL (*p* < 0.001), 0.71 ± 0.06 mg/dL (p <0.001) and 15.07 ± 2.77 mg/dL (p <0.001), 17.17 ± 0.79 mg/dL (*p* < 0.001), respectively (Table 5). Furthermore, the combination of methanol extract with glibenclamide showed serum creatinine and BUN values of 0.73 ± 0.03 mg/dL (*p* < 0.001) and 18.13 ± 2.13 mg/dL (*p* < 0.001) (Figure 5).

**Table 5:** Effect of standard, methanol extract and their combination on creatinine and BUN level after 21 days treatment.

Group	Creatinine (mg/dL)	BUN (mg/dL)
Normal control	0.72 ± 0.09	13.87 ± 2.57
Diabetic control	1.61 ± 0.35	32.93 ± 3.74
Standard	0.73 ± 0.05^∗∗∗^	13.49 ± 3.64^∗∗∗^
MEAR 200	0.71 ± 0.09^∗∗∗^	15.07 ± 2.77^∗∗∗^
MEAR 400	0.71 ± 0.06^∗∗∗^	17.17 ± 0.79^∗∗∗^
MEAR + glibenclamide	0.73 ± 0.03^∗∗∗^	18.13 ± 2.13^∗∗∗^

*Note:* Standard is glibenclamide.

Abbreviation: MEAR, methanol extract of *Annona reticulata* Linn.

^∗∗∗^ *p* < 0.001 (very high significance) against control; Standard is Glibenclamide.

**Figure 6:**
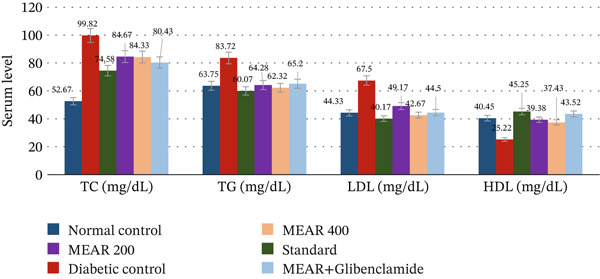
Effect of standard, methanol extract and their combination on lipid profile level after 21 days treatment.

According to Bhowmik et al., excessive levels of small dense LDL particles, low HDL, high TG, and high TC are common characteristics of diabetic dyslipidemia, a term used to describe lipid abnormalities in people with diabetes. Therefore, diabetes may have an impact on the total lipid profile [29]. So, in order to determine whether or not the test samples were able to maintain the lipid profile in diabetic rats, four significant markers of the lipid profile in serum were examined. Serum TC, TG, LDL, and HDL have normal ranges of 10–54 mg/dL, 26–145 mg/dL, 40–50 mg/dL, and 40–50 mg/dL, respectively [30]. Serum TC, TG, LDL, and HDL levels were 52.67 ± 3.90 mg/dL, 63.75 ± 5.63 mg/dL, 44.33 ± 4.50 mg/dL, and 40.45 ± 2.83 mg/dL for the normal control group, and 99.82 ± 44.26 mg/dL, 83.72 ± 2.49 mg/dL, 67.50 ± 7.15 mg/dL, and 25.22 ± 4.43 mg/dL, in due order, for diabetic control group. Consequently, there is a noticeable disturbance in lipid profiling of diabetic control group since the values for TC and LDL are significantly higher and HDL are lower than the normal range. Values for TC, TG, LDL, and HDL were 74.58 ± 7.95 mg/dL (*p* < 0.001), 60.07 ± 8.04 mg/dL (*p* < 0.001), 40.17 ± 4.62 mg/dL (*p* < 0.001), and 45.25 ± 4.81 mg/dL (*p* < 0.001) for the standard group treated with glibenclamide. With the exception of a higher TC value, this group displayed all other values within the normal range. Diabetic rats′ lipid profiles were positively impacted by the methanol extract. Serum levels for TC, TG, LDL, and HDL were 84.67 ± 4.78 mg/dL (*p* < 0.001), 64.28 ± 8.63 mg/dL (*p* < 0.001), 49.17 ± 2.32 mg/dL (*p* < 0.001), 39.38 ± 2.94 mg/dL (*p* < 0.001), and 84.33 ± 3.00 mg/dL (*p* < 0.001), 62.32 ± 3.13 mg/dL (*p* < 0.001), 42.67 ± 3.01 mg/dL (*p* < 0.001), and 37.43 ± 6.47 mg/dL (*p* < 0.001), respectively, for 200 and 400 mg/kg dosages. Group that administered combination of methanol extract and glibenclamide exhibited serum TC, TG, LDL, and HDL levels of 80.43 ± 3.53 mg/dL (*p* < 0.001), 65.20 ± 6.29 mg/dL (*p* < 0.001), 44.50 ± 2.88 mg/dL (*p* < 0.001) and 43.52 ± 3.33 mg/dL (*p* < 0.001) (Table 6). Two hundred and 400 mg/kg dosages of methanolic fraction treated groups demonstrated TG and LDL values within the normal range but TC values were higher and HDL values were lower than the normal range; whereas for the group treated with combination of glibenclamide and methanol extract exhibited all the values within normal range only except higher TC value (Figure 6). Therefore, in diabetic rats, the extract from *A. reticulata*Linn. and its combination showed promising benefits in maintaining the lipid profile.

**Table 6:** Effect of standard, methanol extract and their combination on lipid profile level after 21 days treatment.

Group	TC (mg/dL)	TG (mg/dL)	LDL‐C (mg/dL)	HDL‐C (mg/dL)
Normal control	52.67 ± 3.90	63.75 ± 5.63	44.33 ± 4.50	40.45 ± 2.83
Diabetic control	99.82 ± 44.26	83.72 ± 2.49	67.50 ± 7.15	25.22 ± 4.43
Standard	74.58 ± 7.95^∗∗∗^	60.07 ± 8.04^∗∗∗^	40.17 ± 4.62^∗∗∗^	45.25 ± 4.81^∗∗∗^
MEAR 200	84.67 ± 4.78^∗∗∗^	64.28 ± 8.63^∗∗∗^	49.17 ± 2.32^∗∗∗^	39.38 ± 2.94^∗∗∗^
MEAR 400	84.33 ± 3.00^∗∗∗^	62.32 ± 3.13^∗∗∗^	42.67 ± 3.01^∗∗∗^	37.43 ± 6.47^∗∗∗^
MEAR + glibenclamide	80.43 ± 3.53^∗∗∗^	65.20 ± 6.29^∗∗∗^	44.50 ± 2.88^∗∗∗^	43.52 ± 3.33^∗∗∗^

*Note:* Standard is glibenclamide.

Abbreviation: MEAR, methanol extract of *Annona reticulata* Linn.

^∗∗∗^ *p* < 0.001 (very high significance) against control; Standard is Glibenclamide.

#### 3.2.3. Histopathological Evaluation of Pancreas, Liver and Kidney of Diabetic Rats

**Figure 7:**
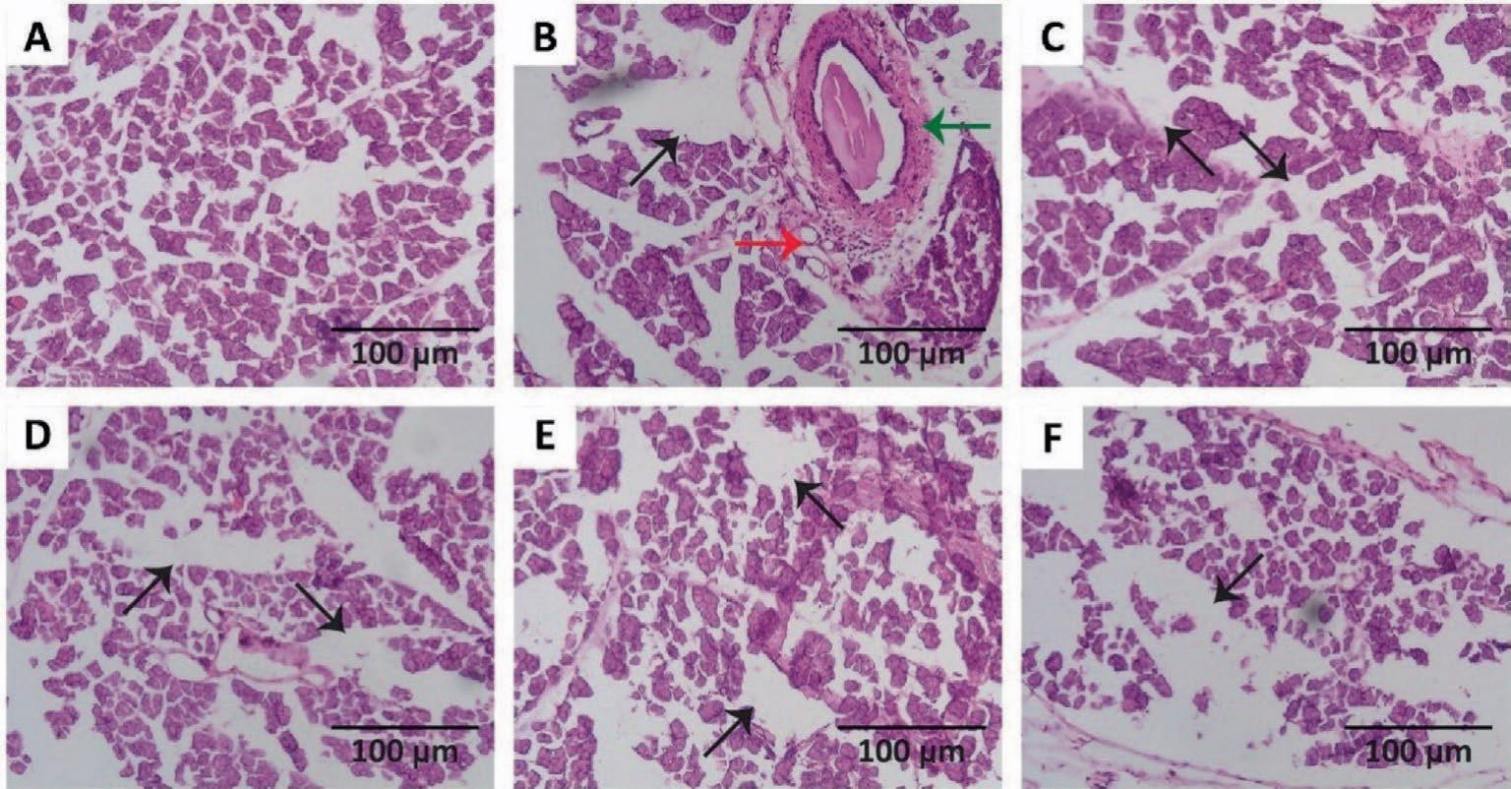
Histopathological evaluation of pancreas, liver, and kidney of diabetic rats. (A) Pancreatic segment of normal control rat, (B) pancreatic section of diabetic rat, (C) pancreatic segment of glibenclamide‐treated diabetic rat, (D) pancreatic section of methanol extract (200 mg/kg)‐treated diabetic rat, (E) pancreatic segment of methanol extract (400 mg/kg)‐treated diabetic rat, and (F) pancreatic section of combination‐treated (both methanol extract and glibenclamide) diabetic rat. In addition, black arrow indicates vacuolation, green arrow indicates inflammatory infiltration, and red arrow indicates necrosis.

Effect of methanol leaf extract and its combination with glibenclamide on pancreatic histopathological profiling in experimental rats (magnification ×20) is shown in Figure 7. Figure 7A shows pancreatic segment of normal control rat demonstrating normal structure. Figure 7B shows slice of Pancreas obtained from diabetic rat depicting damaged islet of langerhans. Figure 7C shows segment of pancreas taken from diabetic rat which administered glibenclamide demonstrating close to natural pancreatic islet of langerhans. Figure 7D shows pancreatic section of diabetic rat which received methanolic fraction at 200 mg/kg dose depicting improved structure of islet of langerhans. Figure 7E shows pancreatic segment collected from diabetic rat which received methanolic fraction at 400 mg/kg dose demonstrating structured islet of langerhans. Figure 7F shows pancreatic slice taken from diabetic rat which administered combination of methanol extract and glibenclamide showing improved structure of islet of langerhans.

**Figure 8:**
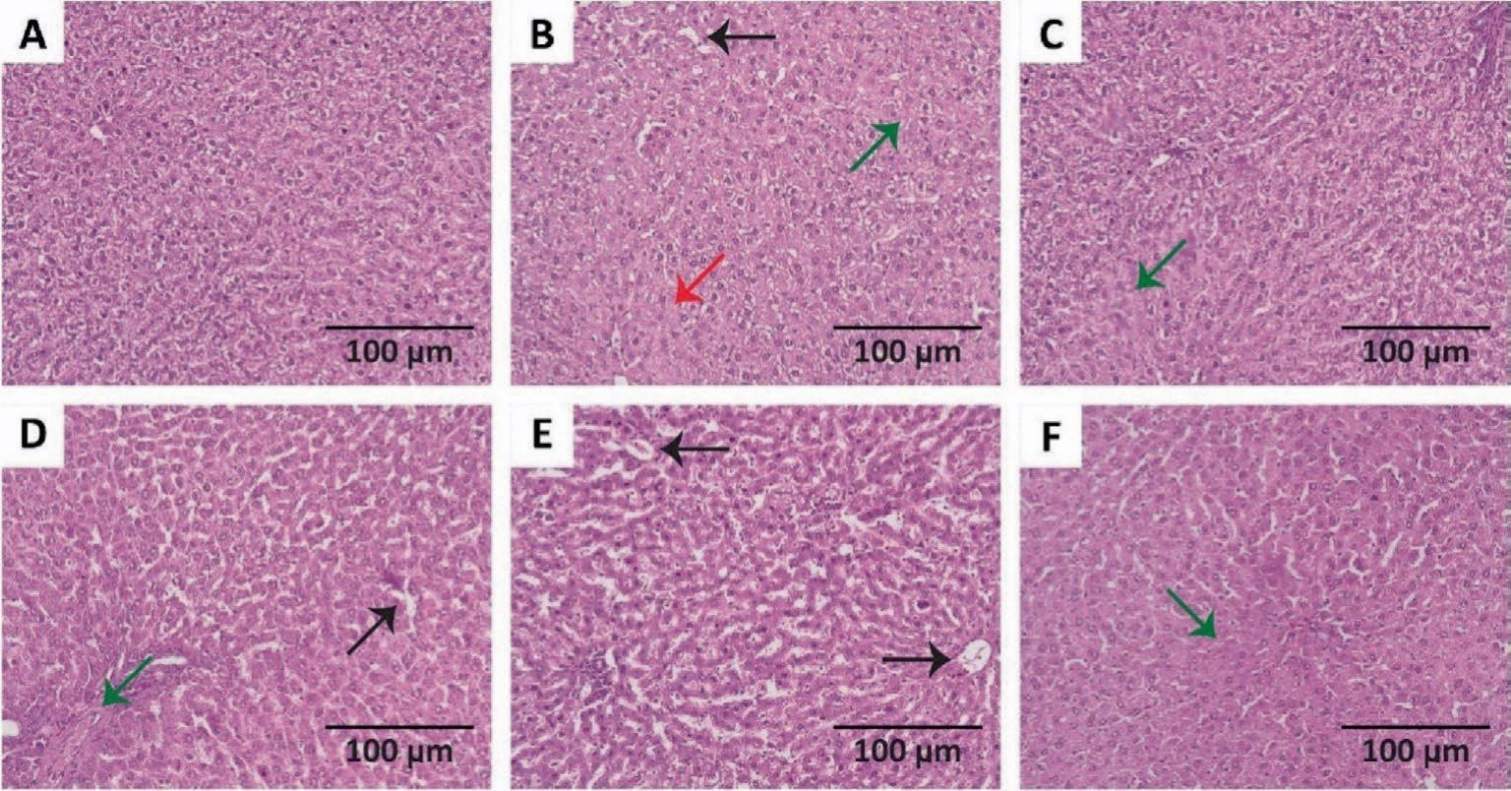
Histopathological evaluation of pancreas, liver, and kidney of diabetic rats. (A) Hepatic segment of normal control rat, (B) hepatic section of diabetic rat, (C) liver segment of glibenclamide‐treated diabetic rat, (D) liver section of methanol extract (200 mg/kg)‐treated diabetic rat, (E) hepatic segment of methanol extract (400 mg/kg)‐treated diabetic rat, and (F) liver section of combination‐treated (both methanol extract and glibenclamide) diabetic rat. Additionally, black arrow indicates vacuolation, green arrow indicates inflammatory infiltration, and red arrow indicates necrosis.

Effect of methanol leaf extract and its combination with glibenclamide on hepatic histopathological profiling in experimental rats (magnification ×20) is shown in Figure 8. Figure 8A shows hepatic segment of normal control rat illustrating normal hepatocytes. Figure 8B shows hepatic slice of untreated diabetic rat demonstrating damaged hepatocytes. Figure 8C liver segment of diabetic rat which was administered glibenclamide demonstrating improved structure of hepatocytes. Figure 8D shows hepatic segment of diabetic rat that was administered 200 mg/kg of methanolic fraction depicting improved structure of hepatocytes. Figure 8E shows section of liver, which was administered 400 mg/kg of methanolic fraction, demonstrated quite arranged hepatocytes. Figure 8F shows liver section of rat administered a combination of methanol extract and glibenclamide illustrating close to natural hepatocytes.

**Figure 9:**
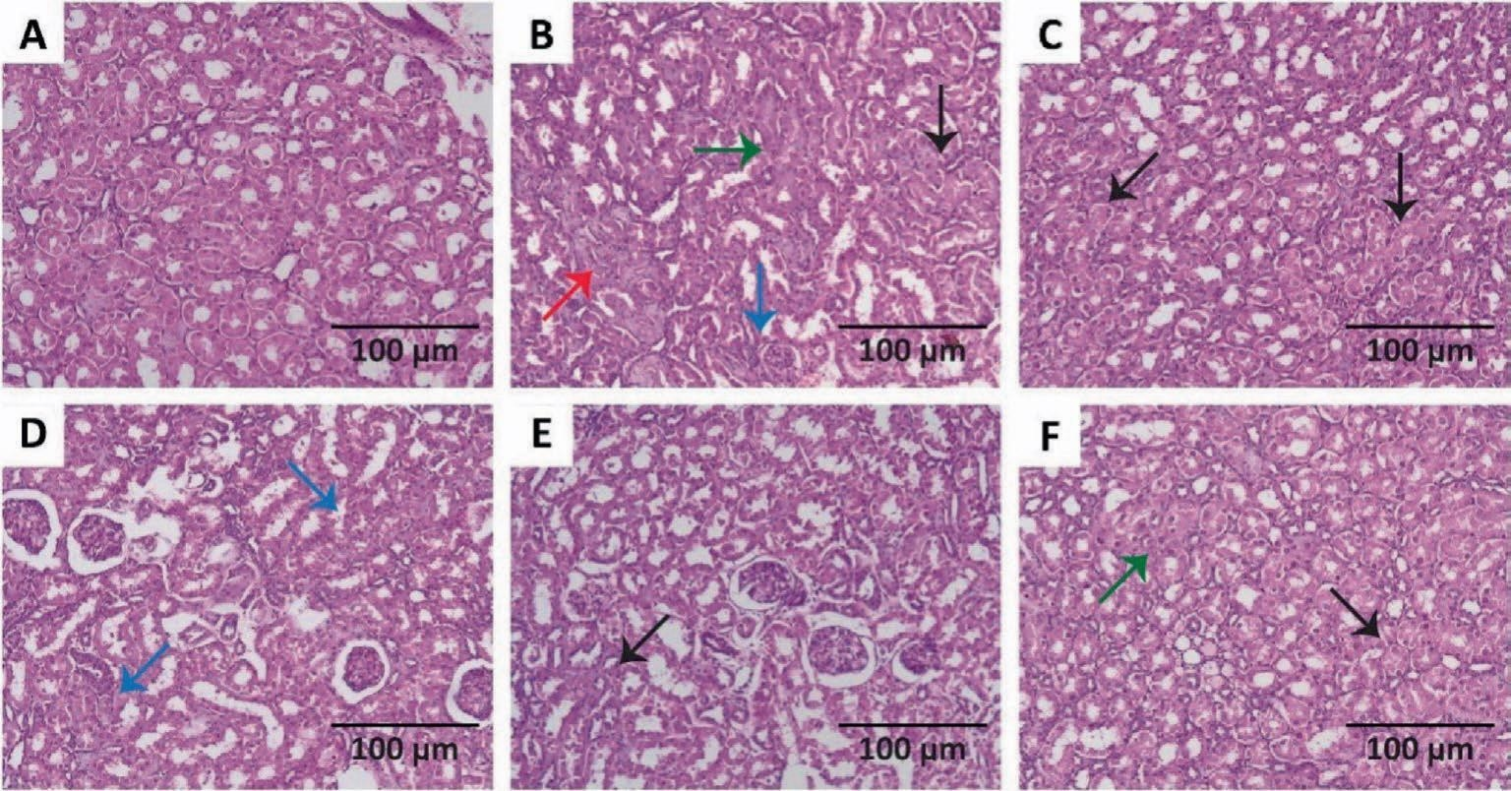
Histopathological evaluation of pancreas, liver, and kidney of diabetic rats. (A) Renal segment of normal control rat, (B) renal section of diabetic rat, (C) kidney segment of glibenclamide‐treated diabetic rat, (D) kidney section of methanol extract (200 mg/kg)‐treated diabetic rat, (E) renal segment of methanol extract (400 mg/kg)‐treated diabetic rat, and (F) kidney section of combination‐treated (both methanol extract and glibenclamide) diabetic rat. Furthermore, black arrow indicates glomerular damage, green arrow indicates inflammatory infiltration, red arrow indicates tubular necrosis, and blue arrow indicates tubular degeneration.

Effect of methanol leaf extract and its combination with Glibenclamide on renal histopathological profiling in experimental rats (magnification ×20) is shown in Figure 9. Figure 9A shows renal section obtained from normal control rat demonstrating normal glomeruli. Figure 9B shows renal segment taken from untreated diabetic rat demonstrating destroyed glomeruli (fat deposition on baseline). Figure 9C shows renal segment collected from diabetic rat that administered glibenclamide depicting improvement in structure of glomeruli. Figure 9D shows renal slice taken from diabetic rat which received methanolic fraction at 200 mg/kg dosage illustrating improved glomeruli structure. Figure 9E shows kidney segment taken from diabetic rat which was administered 400 mg/kg of methanolic fraction depicting close to natural tubules and glomeruli. Figure 9F shows renal slice obtained from diabetic rat which received combination of glibenclamide and methanol extract showing better glomerular structure.

Partial degradation of *β* cells of pancreatic islets is the mechanism by which alloxan causes diabetes, which compromises the amount and quality of insulin these cells generate [31]. However, the alloxan‐induced diabetic model has several limitations. Alloxan selectively destroys pancreatic *β*‐cells through oxidative stress, producing a condition similar to type 1 diabetes rather than Type 2 diabetes, which limits its relevance to the most common clinical form of the disease. Additionally, variability in animal response due to differences in species, strain, dose, and physiological condition may affect the consistency of hyperglycemia and treatment outcomes. Alloxan also exerts nonspecific toxic effects on organs such as the liver and kidneys, which may interfere with metabolic parameters. Despite these limitations, this model is being used by researchers for its cost effectiveness and reliability to produce hyperglycemia through selective pancreatic *β*‐cell destruction [32–34]. The outcomes of the study demonstrated that, methanolic leaf extract of *A. reticulata* Linn. has notable antidiabetic and liver‐renal protective potentials; however, combination of methanolic leaf extract and glibenclamide did not show synergistic action (by comparing to the effects of test extract and drug alone). According to the GC‐MS analytical report, 1,7‐octadien‐3‐ol, 2,6‐dimethyl‐ was detected in the methanol extract. Thesubstance is reported to have significant antidiabetic effect [17]. As per reports, this compound show potential in managing diabetes via glucose regulation, enzyme inhibition, antioxidant effect, improved insulin sensitivity [18]. Additionally, the methanol extract contained hexadecanoic acid‐methyl ester. The hepatoprotective efficacy of the extract and their combination with glibenclamide may be due to the presence of this compound, which is reported to have strong hepatoprotective activity [20]. This compound has promising activity against hepatic dysfunction because it protects the liver by lowering hepatic enzymes, reducing oxidative stress, and promoting hepatocyte regeneration, according to reports [21]. Furthermore, according to GC‐MS analysis, 1,7‐octadien‐3‐ol, 2,6‐dimethyl was detected in the methanol extract. Since the substance is reported tohave renal‐protective impact, it might have contributed to the extract′s kidney‐protective effect [17]. As per reports, this compound provides renal‐protective action by acting as an antioxidant to decrease malondialdehyde levels and increase superoxide dismutase and catalase levels [19]. Moreover, it was discovered that, the methanol extract contained 9‐octadecenamide (z)‐. The compound might help control lipids and cholesterol in diabetic rats since itacts as a potent antihyperlipidemic agent by significantly reducing serum TG, TC, and low‐density lipoprotein cholesterol, according to previous reports [22, 23]. In addition, the aqueous leaf extract of *A. reticulata* Linn. was investigated in our previous study to evaluate its antidiabetic and liver‐renal protective properties. It is noteworthy that the aqueous extract was found to be as effective as the methanolic extract in our current study, indicating the plant’s significant antidiabetic and liver‐renal protective potentials [16]. Furthermore, the antidiabetic potential of extracts from *A. reticulata*Linn. has been studied and verified by some other scientists [35–38]. A few researchers also assessed the hypolipidemic potential of several extracts from *A. reticulata* Linn. [35, 36]. Additionally, several other researchers investigated the hepato‐renal protective effects of *A. reticulata* Linn. extract [39]. This study′s findings are also consistent with those of other researchers. However, for the first time, current investigation identified the probable phytoconstituents of methanolic leaf fraction from *A. reticulata* Linn. that might be responsible for achieving these activities and evaluated the combined effects of methanol extract with glibenclamide in animal model. Although GC–MS analysis indicated the presence of several probable bioactive compounds, these findings are based on tentative identification and do not confirm their direct contribution to the observed pharmacological effects. The identified compounds were not isolated or evaluated individually in the present study. Therefore, further studies involving isolation, purification, and structural confirmation of these constituents, followed by individual pharmacological evaluation, are necessary to verify their bioactivities and to confirm the antidiabetic activity observed in the crude extract.

## 4. Conclusion

*A. reticulata* Linn. leaf extract, both alone and in combination with glibenclamide, demonstrated significant antidiabetic potentials in addition to liver‐renal protective properties in diabetic rats; furthermore, diabetic rats’ lipid profiles were found to be well maintained by these therapies. Therefore, the results of the study suggest that the plant extract from *A. reticulata* Linn. has antidiabetic, anti‐hyperlipidemic, and liver‐renal protective activities. However, the synergistic effects of methanol extract and glibenclamide were not significant. GC‐MS analysis report indicates that test fraction contained a number of components with significant antidiabetic, liver‐renal protective, and anti‐hyperlipidemic properties. So, *A. reticulata* Linn. leaf extract could be an alternative source for the treatment of diabetes mellitus. In addition, definitive conclusion on the current findings may be possible if the bioactive components of the extract are isolated and further tested. This process will aid in understanding mechanism of actions that led to different bioactivities.

## Funding

No funding was received for this manuscript.

## Conflicts of Interest

The authors declare no conflicts of interest.

## Data Availability

The data that support the findings of this study are available from the corresponding author upon reasonable request.
